# Reliability, validity and discriminant ability of the instrumental indices provided by a novel planar robotic device for upper limb rehabilitation

**DOI:** 10.1186/s12984-018-0385-8

**Published:** 2018-05-16

**Authors:** Marco Germanotta, Arianna Cruciani, Cristiano Pecchioli, Simona Loreti, Albino Spedicato, Matteo Meotti, Rita Mosca, Gabriele Speranza, Francesca Cecchi, Giorgia Giannarelli, Luca Padua, Irene Aprile

**Affiliations:** 1 IRCCS Fondazione Don Carlo Gnocchi, Piazzale Morandi 6, 20121 Milan, Italy; 2 Physical Medicine and Rehabilitation Unit, Sant’Andrea Hospital, “Sapienza” University of Rome, Via di Grottarossa, 1035, 00189 Rome, Italy; 3 IRCCS Fondazione Don Carlo Gnocchi, Via di Scandicci, 269, 50143 Florence, Italy; 4 Department of Geriatrics, Neurosciences and Orthopaedics, Università Cattolica del Sacro Cuore, Largo Francesco Vito 1, 00168 Rome, Italy

**Keywords:** Stroke, Upper limb, Robot-mediated evaluation, Reliability, Validity, Discriminant ability

## Abstract

**Background:**

In the last few years, there has been an increasing interest in the use of robotic devices to objectively quantify motor performance of patients after brain damage. Although these robot-derived measures can potentially add meaningful information about the patient’s dexterity, as well as be used as outcome measurements after the rehabilitation treatment, they need to be validated before being used in clinical practice. The present work aims to evaluate the reliability, the validity and the discriminant ability of the metrics provided by a novel robotic device for upper limb rehabilitation.

**Methods:**

Forty-eight patients with sub-acute stroke and 40 age-matched healthy subjects were involved in this study. Clinical evaluation included: Fugl-Meyer Assessment for the upper limb, Action Research Arm Test, and Barthel Index. Robotic evaluation of the upper limb performance consisted of 14 measures of motor ability quantifying the dexterity in performing planar reaching movements. Patients were evaluated twice, one day apart, to assess the reliability of the robotic metrics, using the Intraclass Correlation Coefficient. Validity was assessed by analyzing the correlation of the robotic metrics with the clinical scales, by means of the Spearman’s Correlation Coefficient. Finally, the ability of the robotic metrics to distinguish between patients with stroke and healthy subjects was investigated with t-tests and the Effect Size.

**Results:**

Reliability was found to be excellent for 12 measures and from moderate to good for the remaining 2. Most of the robotic indices were strongly correlated with the clinical scales, while a few showed a moderate correlation and only one was not correlated with the Barthel Index and weakly correlated with the remain two. Finally, all but one the provided metrics were able to discriminate between the two groups, with large effect sizes for most of them.

**Conclusion:**

We found that all the robotic indices except one provided by a novel robotic device for upper limb rehabilitation are reliable, sensitive and strongly correlated both with motor and disability clinical scales. Therefore, this device is suitable as evaluation tool for the upper limb motor performance of patients with sub-acute stroke in clinical practice.

**Trial registration:**

NCT02879279.

**Electronic supplementary material:**

The online version of this article (10.1186/s12984-018-0385-8) contains supplementary material, which is available to authorized users.

## Background

In the last years, Robot – Mediated Therapy has represented one of the most promising approach to restore motor function of upper limb after brain damage [1] mainly because it enables, in comparison with conventional treatment approaches, highly intensive trainings in specifically designed tasks, for extended periods of time [2]. Along with their use as rehabilitation tools, the robotic devices can also act as evaluation tools in order to objectively quantify motor performance of patients after brain damage. In fact, because of their built-in technology in terms of sensors and actuators, the robotic devices are able to acquire data about kinematics and kinetics of patients’ upper limb which are processed to obtain quantitative indices related to the upper-extremity movement quality. According to Sivan et al. [3], these robotic indices are appropriate as a tool to describe bodily functions on all phases of stroke recovery and, therefore, can be effectively used to assess both the level of impairment as well as the improvement after therapy. Robotic indices are therefore increasingly used to assess patients’ dexterity (where loss of dexterity refers to an inability to coordinate muscle activity in the performance of a motor task [4]) with the aim of overcoming, at least partially, the intrinsic limitations of the clinical scale, such as a low rate of reproducibility, low resolution, lack of sensitivity, as well as floor and ceiling effects [5].

Even though most of the studies involve patients with stroke [6–14], robotic evaluations are also used in neurological diseases as Multiple Sclerosis [15], Cerebral Palsy [16, 17], or Ataxia [18].

On their review, Nordin et al. [19] identified more than fifty different kinematic metrics currently used in robot-assisted rehabilitation researches. Usually, the evaluated movement is a reaching task, and more specifically center-out point-to-point movement, since it is important to perform in many activities of daily life. Less often, different tasks, such as shape drawing/tracing tasks, are also analyzed. 

Although these new robot-derived measures can potentially add meaningful information about the patient’s performance, their properties in terms of reliability, validity and responsiveness should be assessed, before their use in clinical practice. In fact, in order to be brought into the clinical field, they have to be stable, sensitive and clinically meaningful measures. The review of Maciejasz et al. [20] identified more than 120 robotic devices for upper limb rehabilitation and most of them allow measuring kinematic and/or kinetic parameters which describe the motor ability of patients. If one considers the amount of robots for the upper limb that are currently available, few studies have investigated the psychometric properties of the robotic indices [7, 18, 21] and, except for a few cases, a complete analysis of their metric characteristics and concurrent validity with clinical scales is missing [19]. In addition, it is mandatory to validate the metrics provided by the specific device of interest. In fact, the robotic structure and the provided support can be different among devices, affecting the validity and sensitivity of the results [7]. As suggested by Nordin et al. [19], the mechanical structure of the robot, as well as its control scheme, play an important role in providing assessment data. As an example, data obtained from end-effector robots cannot be directly compared with those provided by exoskeletons, since the degree of interaction between patients and robot is different in terms of support and mechanical interface and this could affect the patient’s performance. The results obtained with a specific device cannot be arbitrarily extended to a different one, since they likely have a different conception. Therefore, for each device it is necessary to verify the validity and sensitivity of the instrumental outcome measures.

Recently, a novel type of haptic interface was proposed, which is fully portable and employs onboard sensors and electronics to solve accurate localization and also uses motors for force feedback generation [22]. This end-effector device has been designed for application in neuro-rehabilitation protocols and it adopts specific mechanical, electrical and control solutions in order to cope with patient requirements. Along with several therapeutic scenarios, it also qualifies as an evaluation tool providing some indices about the patients’ sensor-motor skills, similar to those already described in literature.

To the best of our knowledge, however, the quantitative indices provided by this device have not yet been validated in terms of their psychometric properties. Therefore, the goal of the present work is to evaluate, within a multicenter study aimed to compare a traditional and a robotic rehabilitation approach, the reliability, the concurrent validity and the discriminant ability of the indices provided by a novel rehabilitation device during an unassisted reaching task.

## Methods

### Participants

Forty-eight consecutive patients with subacute stroke (both inpatient and outpatients) were enrolled in 4 different rehabilitation centers of the Fondazione Don Carlo Gnocchi for this study. Inclusion criteria were: (1) first-ever stroke (cerebral infarction or hemorrhage), confirmed by either brain CT or MRI findings (2) age between 40 and 85 years; (3) time latency since stroke ranging from 2 weeks to 6 months; (4) cognitive and language abilities sufficient to understand the experiments and follow instructions. Exclusion criteria were: (1) upper extremity Fugl-Meyer score > 58; (2) behavioral and cognitive disorders and/or reduced compliance that would interfere with active therapy; (3) fixed contraction deformity in the affected limb that would interfere with active therapy (ankylosis, Modified Ashworth Scale = 4); (4) inability to discriminate distinctly the images showed on a 22″ monitor placed at the eye level of each subject at a distance of about 50 cm, even with corrective glasses. Forty age-matched subjects without neurological or other relevant medical conditions served as a reference population. Demographic and characteristics of the participants are shown in Table 1.

**Table 1:** Demographic and clinical characteristics of the sample

	Patients with stroke (*n* = 48)	Healthy subjects (*n* = 40)
Sex M/F	33/15	26/14
Age, mean ± SD (years)	64 ± 11	65 ± 13
Classification		
Cerebral ischemia (N)	28	–
Cerebral hemorrhage (N)	20	–
Time from lesion, mean ± SD (days)	88 ± 42	–
FMA-UL, mean ± SD	29 ± 18	–
ARAT, mean ± SD	15 ± 18	–
BI, mean ± SD	40 ± 24	–

*SD* Standard Deviation, *FMA-UL* Fugl-Meyer Assessment for the Upper Limb, *ARAT* Action Research Arm Test, *BI* Barthel Index

This study is a cross-sectional objective analysis of baseline data collected as part of a larger clinical trial, approved by the institutional ethics committee (FDG_6.4.2016) and registered at clinicaltrials.gov with identifier number (NCT02879279). All participants gave informed consent according to the Declaration of Helsinki.

### Clinical assessment

Patients were clinically evaluated using the upper limb part Fugl-Meyer Assessment of Motor Recovery after Stroke (FMA), the Action Research Arm test (ARAT) and the Barthel Index (BI).

The FMA evaluates recovery in post-stroke hemiplegic patients and it is one of the most widely used quantitative measures of motor impairment [23]. It is characterized by a high inter-rater reliability [24, 25] and validity [26]. This measure includes five domains (motor function, sensory function, balance, joint range of motion, joint pain) to assess synergistic and voluntary movement after stroke. A three-point ordinary scale is used to assess movement (0 = unable; 1 = partial; 2 = performs fully) in each item. In this research we used the upper limb section in the motor function domain (FMA-UL). The score ranges from 0 (most severe impairment) to 66 (no impairment).

The ARAT [27] assesses upper limb function using observational methods and consists of 19 items organized in 4 sections: Grasp, Grip, Pinch and Gross movements. The performance of each task is scored on a 4-point ordinal scale (0 = unable to complete any part of the task, 1 = the task is only partially completed, 2 = the task is completed but with great difficulty and/or in an abnormally long time, and 3 = the movement is performed normally). The maximum ARAT score is 57 points, which means normal upper limb function.

The BI [28] assesses the ability of an individual with a neuromuscular or musculoskeletal disorder to take care of him/herself, and consists of 10 items, evaluating both personal care (feeding, dressing, hygiene) and mobility activities (transferring, walking/wheeling). Possible values range from 0 to 100, with lower scores representing greater dependency.

### Equipment and robotic assessment

**Fig. 1:**
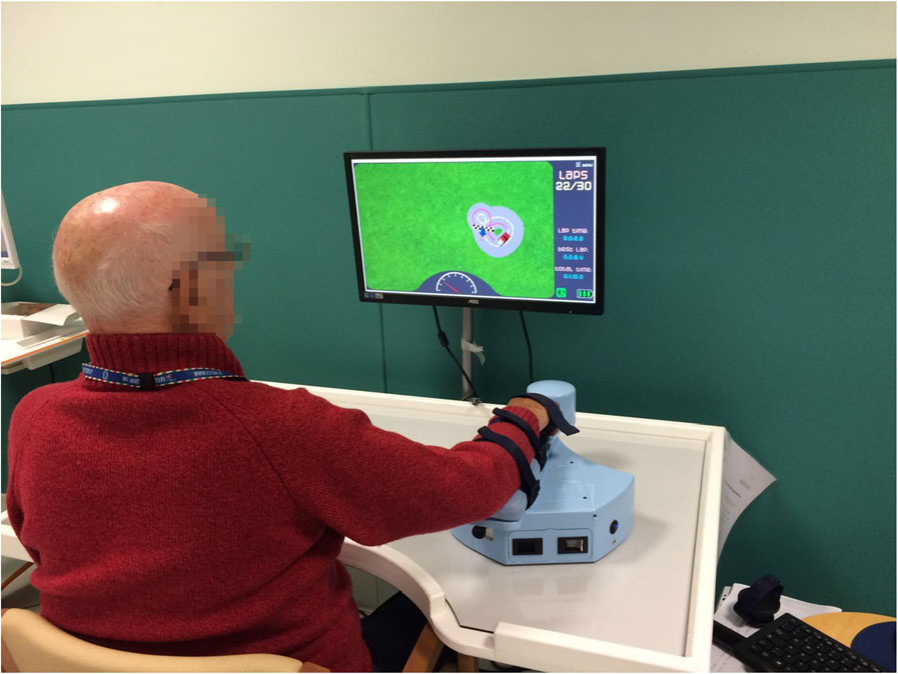
Patient engaged in a rehabilitation session with MOTORE

The robotic assessment of upper limb motor performance was conducted by means of MOTORE (MObile roboT for upper limb neurOrtho Rehabilitation, Humanware, Italy), see Fig. 1. This is a planar end-effector device designed for application in neuro-rehabilitation protocols and it adopts specific mechanical, electrical and control solutions in order to meet the requirements of neuro-rehabilitation. MOTORE is equipped with an onboard computing unit, an odometry system (based on encoders) and a specifically designed global localization system (which recognizes patterns on the working surface). In fact, the device moves by means of transwheels on the planar working surface and it uses a 2DOF load cell in the handle to measure the interaction force with the patient. The device has 3 DC motors so that it can (a) help the patient when he/she is not able to accomplish the task, (b) prevent movements different from the ideal trajectories, (c) provide different weight and viscosity behaviors, (d) maintain a proper orientation on the plane. The device generates force feedback without any intermediate link to the ground or frame, thanks to the motion of the wheels and using the information obtained from the load cell. A Bluetooth connection links the device to a PC unit, where a software shows targets to be reached and trajectories to be followed as well as a user/therapist interface for the selection of the exercise parameters. The robot is controlled in admittance mode: forces measured by the load cell are used to determine the linear velocity of the device, on the basis of two parameters (*M*, that is the apparent mass of the device, and *b*, that is the nominal viscosity) that can be modified to change the robot behavior [29]. Compared with other similar robotic systems, it is characterized by its portability, being specifically conceived for teleoperation applications. During the rehabilitation session, ambulatory subjects are comfortably seated on a chair, while non-ambulatory patients are seated on their wheelchair, in front of a height-adjustable table. The center of the workspace is located in front of the subject at the midline of the body. Subject’s forearm is supported by the device, with his/her hand grasping the handle of the robot.

**Fig. 2:**
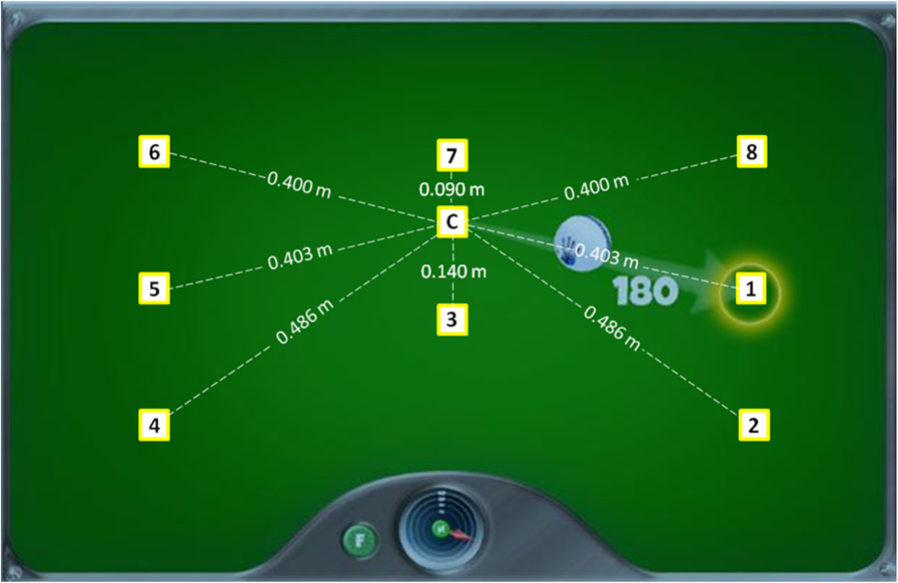
The evaluation task of MOTORE. In figure is showed the visual feedback showed to the patients on the screen, together with the position of each target. The white ball indicates the position of the end-effector; the yellow circle indicates the target to be reached. The yellow squares, not showed to the patient during the task, indicate the position of the targets: C is the central target, while the numbers from 1 to 8 indicate the external targets with the sequence of the center-out movements. In addition, the distance of each target from the center is reported

Similar to other devices, together with several rehabilitation exercises (based both on tracking or occupational-like exercises) it provides an Evaluation Task, based on a center-out point-to-point reaching activity: following a visual feedback, subjects are asked to move the device from the center to a peripheral target and come back to the center, starting at the “East” position and proceeding clockwise, making a total of 16 reaching movements. During the Evaluation Task, both the position of the robot (a white ball) and of the target to be reached (a yellow circle) are shown on the screen. The provided visual feedback, the target location and the movement sequence are shown in Fig. 2. Once the test is completed, several indices are computed by the device and displayed to give a feedback to the patient about her/his performance. These indices are summarized in Table 2. During the Evaluation Task, the apparent mass *M* and nominal viscosity *b* are set to the minimum, to minimizing the inertia of the device and, therefore, to allow the patient to move it with the least possible effort.

**Table 2:** Outcome measures provided by MOTORE

Index	Description
Duration	Time required to complete the task
Velocity_mean_	Average velocity of the device during the test
Length_tot_	Global length of the path travelled by the subject during center-out movements; it ranges from 0 (no movement) to 2.808 m (patient can fully perform the entire task)
Length_i_	Length of the path travelled by the subject toward the i-th target (*i* = 1:8)
Score	Mean of the ratios between the actual distance covered by the patients and the required distance to be travelled, computed for each required movement. It ranges from 0 (no movement) to 10 (the patient can fully perform the required task)
Work_tot_	Line integral of the force along the path described by the patient
Work_tan_	The amount of total work directed towards the target

### Experimental protocol

In our study, each participant was asked to perform the Evaluation Task provided by the device three times, making a total of 48 reaching movements (i.e., three nonconsecutive reaching movements for each direction). The participants were not asked to perform the task with a specific time constraint and, then, the movement accuracy was implicitly a task requisite [18, 30]. When a patient or a healthy subject was unable to reach a target (due to the upper limb impairment, or to the wide investigated workspace), he/she was asked to move the robot as far as possible toward the target. For each subject, a session (three repetitions of the Evaluation Task) lasted between 5 and 10 min, depending on the patient’s impairment.

All the patients and a subgroup of healthy subjects were tested twice, 1 day apart, to assess the test-retest reliability of the provided outcome measures. For both test sessions, the value of each metric obtained in the three repetition was recorded and their mean value was computed and used for the statistical analysis.

### Statistical analysis

All statistical analyses were performed using MedCalc (version 17, MedCalc Software, Ostend, Belgium) and SPSS (version 20, SPSS Inc., Chicago IL, USA).

#### Test-retest reliability

Relative test-retest reliability was assessed based on data obtained from patients at the two test sessions by using the Intraclass Correlation Coefficient (ICC), using a two-way random effect, absolute agreement, multiple measurements model. Reliability was classified as excellent (ICC > 0.90), good (0.75 < ICC ≤ 0.90), moderate (0.5 < ICC ≤ 0.75) or poor otherwise [31]. Absolute test-retest reliability was analyzed comparing for each index data obtained during the two test sessions by mean of paired t-tests and Bland-Altman plots.

Intra-session reliability was investigated in stroke patients comparing the data obtained in the three repetitions, for each session separately, by using a repeated measure ANOVA test. For each index, if the test was significant, a post-hoc analysis with Bonferroni correction was carried out.

#### Concurrent validity

To assess the concurrent validity of the robotic indices, the correlations between the robotic parameters and the clinical scales (FMA-UL and ARAT) were investigated using the Spearman’s rank correlation coefficients. The same analysis was used to investigate the relationships between robotic indices and impairment in the activities of daily living, as measured by the BI. The coefficient values were interpreted as follows [32]: 0.0–0.2 little if any; 0.2–0.4 weak; 0.4–0.7 moderate; 0.7–1.0 strong.

#### Discriminant ability

The ability of the robotic indices to discriminate stroke patients from healthy subjects was evaluated by means of unpaired t tests; for each index, the effect size was also evaluated through the Cohen’s d coefficient (small ≥0.20, medium ≥0.50, large ≥0.80 [33]).

For all the statistical analysis, a *p* value less than 0.05 was deemed significant.

## Results

### Test-retest reliability

ICCs and 95% confidence intervals, as well as the results of the statistical analysis of the comparison of the two assessments, are shown in Table 3.

**Table 3:** Test-retest reliability in stroke patients (*n* = 48)

	Test mean (SD)	Retest mean (SD)	ICC	95% CI		Paired t test (P)
				Lower bound	Upper bound	
Duration (s)	193.7 (107.30)	176.8 (111.90)	0.962	0.922	0.980	**0.004**
Velocity_mean_ (m/s)	0.05 (0.04)	0.07 (0.04)	0.914	0.756	0.962	**< 0.001**
Length_tot_ (m)	1.80 (0.92)	1.84 (1.01)	0.951	0.912	0.972	0.495
Lenght_1_ (m)	0.25 (0.16)	0.25 (0.18)	0.930	0.876	0.960	0.784
Lenght_2_ (m)	0.38 (0.14)	0.35 (0.17)	0.804	0.652	0.890	0.149
Lenght_3_ (m)	0.13 (0.03)	0.13 (0.02)	0.693	0.456	0.828	0.542
Lenght_4_ (m)	0.34 (0.18)	0.36 (0.17)	0.917	0.851	0.953	0.113
Lenght_5_ (m)	0.24 (0.17)	0.25 (0.18)	0.907	0.834	0.948	0.551
Lenght_6_ (m)	0.20 (0.17)	0.22 (0.18)	0.917	0.852	0.953	0.13
Lenght_7_ (m)	0.05 (0.04)	0.06 (0.04)	0.957	0.924	0.976	0.188
Lenght_8_ (m)	0.21 (0.17)	0.22 (0.18)	0.934	0.883	0.963	0.467
Score	7.91 (2.20)	7.99 (2.45)	0.972	0.949	0.984	0.477
Work_tot_ (J)	19.88 (12.75)	20.79 (15.45)	0.908	0.837	0.949	0.446
Work_tan_ (J)	10.22 (8.65)	11.26 (0.18)	0.957	0.922	0.976	0.061

Intraclass Correlation Coefficient (ICC) with 95% Confidence Interval (CI), and result of the t tests. Bold values indicated statistical significance, with *p* value less than 0.05

**Fig. 3:**
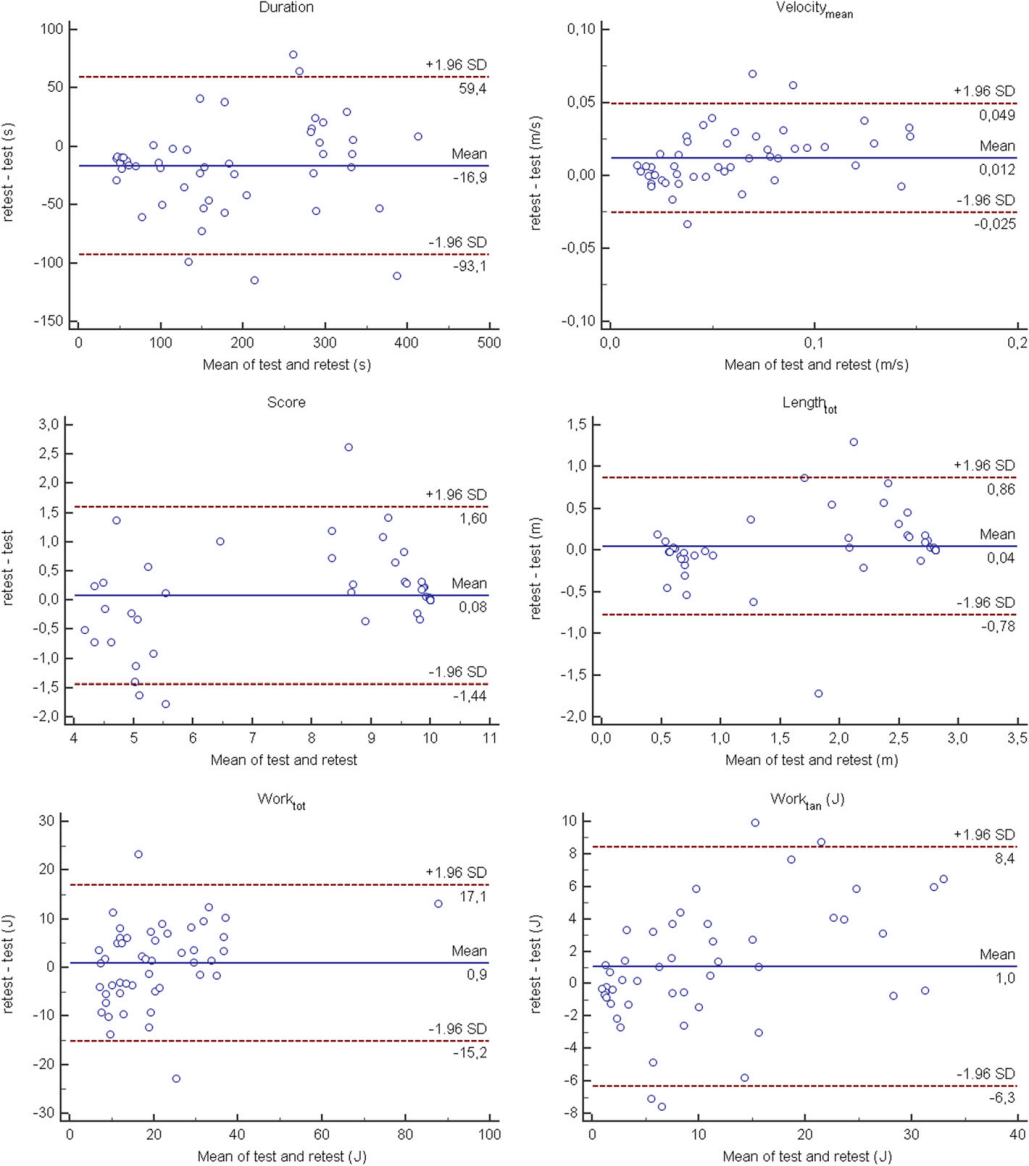
Bland-Altman plots of the robotic indices assessing the whole task

**Fig. 4:**
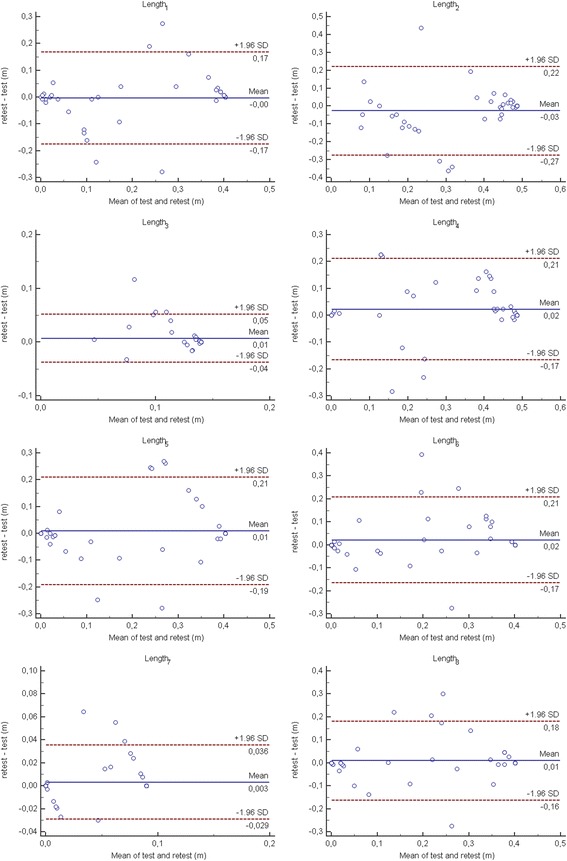
Bland-Altman plots of the robotic indices assessing the path length travelled by stroke patients towards each target

Referring to the relative test-retest reliability, Duration, Velocity_mean_, Length_tot_, Length_1_, Length_4_, Length_5_, Length_6_, Length_7_, Length_8_, Score, Work_tot_ and Work_tan_ displayed an excellent reliability (ICC > 0.9), while a good (ICC ≥ 0.75) and a moderate (ICC ≥ 0.5) reliability was shown by Length_2_ and Length_3_ respectively. With respect to the absolute reliability, we found a statistically significant reduction of Duration (*p* = 0.004) and a statistically significant increase of Velocity_mean_ (*p* < 0.001), when data obtained at the first test session were compared with those obtained 1 day after (see Figs. 3 and 4 for Bland-Altman analysis).

**Fig. 5:**
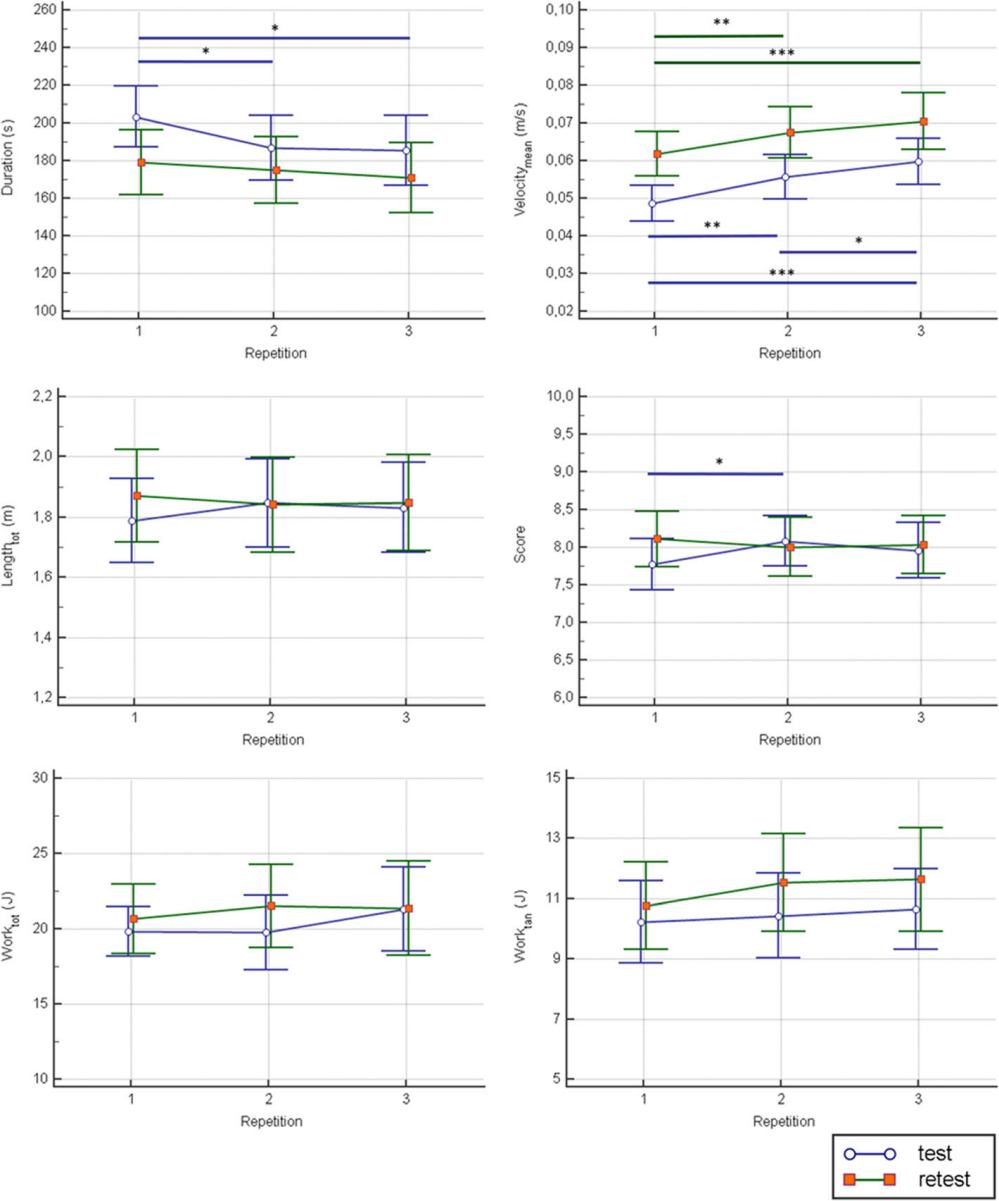
Intra-session reliability analysis in stroke patients: robotic indices assessing the whole task. Blue lines represent the statistical analysis of the first session (test), while green lines represent the statistical analysis of the second session (retest). The symbols *, ** and *** represent a statistically significant difference between repetitions, with a *p* value less than 0.05, 0.01 and 0.001, respectively

**Fig. 6:**
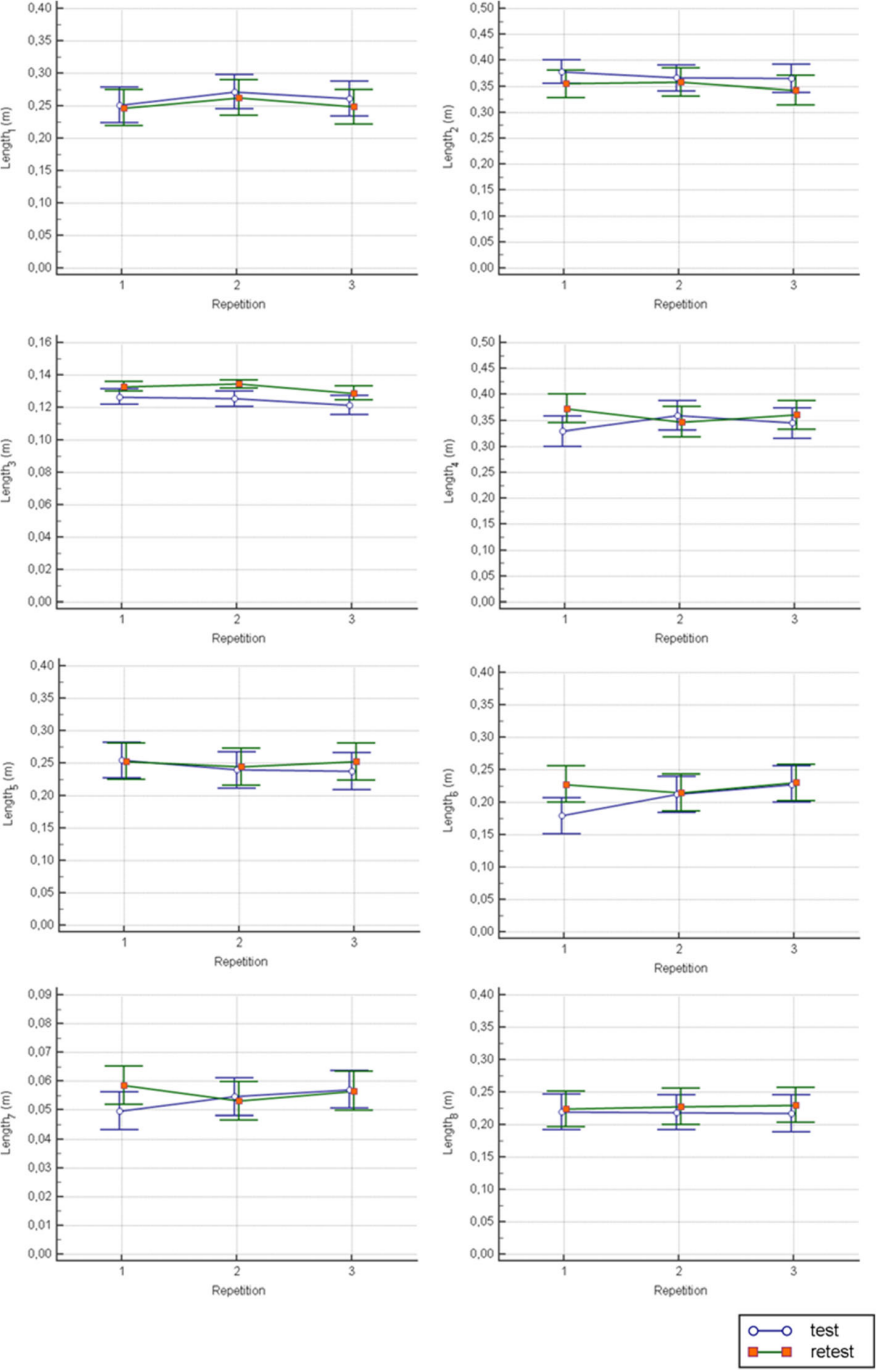
Intra-session reliability analysis in stroke patients: robotic indices assessing the path length travelled towards each target. Blue lines represent the statistical analysis of the first session (test), while green lines represent the statistical analysis of the second session (retest)

Finally, the intra-session reliability showed, during the test, a significant decrease of the Duration (*p* = 0.05), and a significant increase of the Velocity_mean_ (*p* < 0.001) and the Score (*p* = 0.045), while, during the retest, only a significant increase of the Velocity_mean_ was found (*p* = 0.001). With respect all the remaining indices, no differences between repetitions were found (see Figs. 5 and 6).

With respect to the healthy subjects, we found that the relative test-retest reliability was excellent for the Duration, good for the Velocity_mean_ and the Work_tan_, and moderate to poor for all the remaining indices (Table 4). The absolute reliability showed that a significant decrease of the Duration (p < 0.001) and a significant increase of the Velocity_mean_ (*p* = 0.014) and the Work_tan_ (*p* = 0.04).

**Table 4:** Test-retest reliability in healthy subjects (*n* = 19)

	Test mean (SD)	Retest mean (SD)	ICC	95% CI		Paired t test (P)
				Lower bound	Upper bound	
Duration (s)	107.3 (56.30)	83.96 (50.02)	0.914	0.336	0.977	**0.000**
Velocity_mean_ (m/s)	0.08 (0.04)	0.10 (0.06)	0.81	0.437	0.931	**0.014**
Length_tot_ (m)	2.64 (0.29)	2.68 (0.21)	0.593	−0.064	0.844	0.484
Lenght_1_ (m)	0.39 (0.03)	0.39 (0.03)	0.627	−0.002	0.858	0.966
Lenght_2_ (m)	0.46 (0.08)	0.47 (0.09)	0.93	0.822	0.973	0.309
Lenght_3_ (m)	0.14 (0.01)	0.14 (0.00)	§	§	§	0.331
Lenght_4_ (m)	0.46 (0.10)	0.48 (0.01)	0.01	−1.511	0.615	0.273
Lenght_5_ (m)	0.38 (0.06)	0.39 (0.03)	0.087	−1.446	0.652	0.458
Lenght_6_ (m)	0.35 (0.07)	0.35 (0.09)	0.722	0.260	0.894	0.870
Lenght_7_ (m)	0.09 (0.01)	0.09 (0.00)	§	§	§	0.358
Lenght_8_ (m)	0.38 (0.04)	0.38 (0.06)	0.814	0.512	0.929	0.729
Score	9.68 (0.58)	9.78 (0.37)	0.522	−0.249	0.816	0.453
Work_tot_ (J)	17.62 (10.29)	19.84 (8.21)	0.695	0.227	0.882	0.296
Work_tan_ (J)	13.17 (7.38)	15.42 (6.61)	0.868	0.632	0.950	**0.040**

Intraclass Correlation Coefficient (ICC) with 95% Confidence Interval (CI), and result of the t tests. Bold values indicated statistical significance, with *p* value less than 0.05. The symbol § indicate null variance in the data

### Concurrent validity

The results of the correlation analysis between the robotic indices and the clinical scale are shown in Table 5. Most of the robotic indices showed a strong correlation with the FM, with Length_2_ e Length_3_ being moderately correlated and Work_tot_ weakly correlated with the FM. When examining correlations between robotic indices and the ARAT, we observed similar results to those obtained with the FM, with slightly lower correlation coefficients overall. Finally, all the provided indices but the Work_tot_ were moderately correlated (11 indices) to strongly correlated (2 indices, namely Length_tot_ and Score) with the BI. It is worthy to note that almost all the correlations are significant wit a p level lower than 0.001 and, therefore, they remain significant even after a Bonferroni correction (i.e., with an alpha set to 0.05/42 = 0.0012, where 42 is the number of analyzed correlations). The results of the correlation analysis between the robotic indices are provided as Additional file 1: Table S1.

**Table 5:** Validity

	FMA-UL	ARAT	BI
Duration	−0.8507^***^	−0.7716^***^	− 0.6738^***^
Velocity_mean_	0.8227^***^	0.7587^***^	0.6340^***^
Length_tot_	0.8551^***^	0.7268^***^	0.7139^***^
Lenght_1_	0.7700^***^	0.6273^***^	0.6423^***^
Lenght_2_	0.6259^***^	0.5904^***^	0.4419^**^
Lenght_3_	0.5093^***^	0.4613^***^	0.4195^**^
Lenght_4_	0.7172^***^	0.5689^***^	0.6021^***^
Lenght_5_	0.8047^***^	0.6666^***^	0.6638^***^
Lenght_6_	0.8026^***^	0.7075^***^	0.6914^***^
Lenght_7_	0.8267^***^	0.6979^***^	0.6404^***^
Lenght_8_	0.8584^***^	0.7196^***^	0.6241^***^
Score	0.8443^***^	0.7384^***^	0.7417^***^
Work_tot_	0.3472^**^	0.3672^**^	0.1700
Work_tan_	0.8188^***^	0.6942^***^	0.6564^***^

Spearman’s correlation coefficient between the robotic indices and the following clinical scale: Upper limb subscale of the Fugl-Meyer Assessment (FMA-UL), Action Research Arm Test (ARAT), and Barthel Index (BI). The symbols **, and *** indicate a *p* value less than 0.01 and 0.001 respectively

### Discriminant ability

The expected ability of the robotic indices to distinguish between patients with subacute stroke and age-matched healthy subjects was confirmed by the results of the statistical analysis. In fact, all the robotic indices but the Work_tot_ obtained from patients with sub-acute stroke were statistically different from those of controls (see Table 6). The analysis of the effect size showed that the discriminant ability was medium for the Work_tan_ and large for all the remaining indices, being ES higher than 1 for 8 of them.

**Table 6:** Discriminant ability

Robotic indices	Patients with stroke (*N* = 48) Mean (SD)	Healthy subjects (*N* = 40) Mean (SD)	Unpaired t test (P)	Effect Size
Duration (s)	193.7 (107.30)	93.30 (53.50)	< 0.001	1.18
Velocity_mean_ (m/s)	0.05 (0.04)	0.09 (0.05)	< 0.001	0.86
Length_tot_ (m)	1.80 (0.92)	2.67 (0.24)	< 0.001	1.30
Lenght_1_ (m)	0.25 (0.16)	0.39 (0.03)	< 0.001	1.17
Lenght_2_ (m)	0.38 (0.14)	0.46 (0.08)	0.001	0.75
Lenght_3_ (m)	0.13 (0.03)	0.14 (0.01)	0.004	0.62
Lenght_4_ (m)	0.34 (0.18)	0.47 (0.07)	< 0.001	0.98
Lenght_5_ (m)	0.24 (0.17)	0.39 (0.04)	< 0.001	1.20
Lenght_6_ (m)	0.20 (0.17)	0.35 (0.08)	< 0.001	1.17
Lenght_7_ (m)	0.05 (0.04)	0.09 (0.00)	< 0.001	1.31
Lenght_8_ (m)	0.21 (0.17)	0.38 (0.05)	< 0.001	1.33
Score	7.91 (2.20)	9.74 (0.47)	< 0.001	1.16
Work_tot_ (J)	19.88 (12.75)	19.03 (9.11)	0.717	–
Work_tan_ (J)	10.22 (8.65)	14.62 (6.98)	0.010	0.56

Descriptive statistics for the robotic indices in patients with stroke (*N* = 48) and healthy subjects (*N* = 40). Comparison is assessed by means of t tests. For significant differences, the Effect Size is also reported

## Discussion

In this study we assessed for the first time the intra-session and the between-day test-retest reliability, and the validity of the outcome measures provided by a novel planar robot for upper limb rehabilitation, in a sample of patients with sub-acute stroke, and their ability to differentiate patients from a group of age-matched healthy subjects. The abovementioned outcome measures assess the ability of patients in performing a planar reaching task. Similar protocols are provided by several robotic devices and extensively used to assess the residual motor ability of the upper limb in patients with stroke [6–14], or other neurological diseases [15, 16, 34]. However, the specific mechanical, electrical and control solutions adopted in the device requires a validation of the provided measures, since the results obtained from different devices cannot be simply extended [7]. In fact, because each robot differ from the others in terms of provided support, mechanical structure and control algorithm, the validity and the sensitivity of similar metrics could be different among different devices [7].

Differently from clinical scales, that are worldwide recognized and easy to administered in any rehabilitation center, robotic outcome measures can be used only in center equipped with similar devices, and the obtained results are hard to share among centers. However, the metrological characteristic of these measures are often superior to those of clinical scales and, therefore, they can be a very powerful tool to monitor the improvement of the patients, at least in centers where similar devices are installed. Moreover, the increasing data sharing capacity, as well as the spread of these devices, may improve in the future diffusion and use of these data among centers.

With respect to the relative reliability, as assessed by the ICCs, we found that almost all the provided indices exhibited good to excellent reliability across the two separate testing days, in patients with sub-acute stroke. These results are in accordance with previous works, where a high reliability was shown by similar indices provided by other upper limb robotic devices [8, 13, 35] in stroke patients. It is worth noting that several indices showed an ICC value higher than 0.9, meaning that they could be used for intra-individual comparisons (i.e. for individual decision-making) and not just for group-level comparisons (i.e. for the evaluation of a whole large group of patients), where an ICC value of 0.7 level is acceptable.

With respect to the absolute reliability, an unexpected result was the significant decrease of the duration and the significant increase of the Velocity_mean_ in the second evaluation (retest), when compared with the first (test). It is likely that in the first test session patients were more cautious in performing the required task, moving the robot in a slower way, if compared to the second test session. These results would have probably been different if patients had performed a practice test before the first evaluation, in order to familiarize with the device. In fact, it must be highlighted that we have deliberately chosen not to perform a practice test before the first evaluation. Analyzing the data coming from each repetition in the first day of evaluation, we found a significant trend in both indices that, in the second day was absent for the Duration and less evident for the Velocity_mean_. Therefore, our results support the hypothesis that, at least with respect to these two indices (Duration and Velocity_mean_), in clinical practice as well as in research study, some familiarization trials, before the actual evaluation, should be performed. This is particularly true because both Duration and Velocity_mean_ are hallmarks of the upper limb impairment following a stroke [36] and they have to be evaluated in a robotic assessment.

On the contrary, no other indices showed significant differences in the two evaluations confirming their absolute reliability, meaning that patients did not change the travelled path or the mechanical work produced to move the hand/robot.

With respect to the healthy subjects, similar or slightly lower ICC values were found for the indices independent from the travelled distance (i.e., the Duration, the Velocity_mean_ and the Work_tan_), while we obtained very low ICC values for almost all the metrics related to the travelled distance. This can be easily explained with the very low to null between-subject variance in the data. Similar to the stroke patients, a learning effect was detected, as showed by the statistical significant differences in Duration, Velocity_mean_ and Work_tan_ between the two evaluations.

The validity study showed that all investigated indices were significantly correlated with the Fugl-Meyer assessment and the Action Research Arm Test. This led us to confirm the concurrent validity of the robotic indices against common clinical scale of upper limb impairment, implying that they provide meaningful information from a clinical point of view. Compared to the clinical scales, the robotic assessment can be obtained quickly and recorded at several time-points during the rehabilitation path. The relation between the FM and the robotic assessment has been largely studied, being the FM the most commonly used clinical scale used in trial involving robotic devices [3]. Generally, the robotic indices were found to be correlated with the FM with similar or lower correlation coefficient [5, 7, 10, 11, 21, 37–39], when compared with those obtained with MOTORE. Similar results were found in the correlation with the ARAT. This result is not surprising, since the FM and the ARAT were found to be highly correlated to each other [40, 41]. The correlation coefficients we found were generally higher, when compared to other studies [42]. A possible explanation could be the greater variability in patient’s disability in our study, when compared to that of other studies (see, for example, [7, 12, 37]). In fact, it is known that the value of the correlation coefficient is greater if there is more variability among the observations [43]. Of particular interest is the result about the correlation between the robotic measures and the BI, being the BI a global measure of disability rather than a motor assessment scale. This means that the upper limb motor performance, even if measured in a simple planar reaching task but in instrumental way, could, at least partially, reflect the ability in the activities of daily living.

The differences we have found between the different directions in terms of validity can be related to the different level of difficulty of the required movement. In fact, higher correlation coefficients were found for the movements towards the targets farther from the subject’s body (i.e., 6, 7 and 8), while lower coefficients were found for the movements towards the targets nearer the subject’s body (i.e., 2, 3 and 4). These differences can be explained by considering some clinical aspects about the upper limb motor recovery in patients with stroke. In most cases, stroke patients are facilitated to perform flexion elbow movements and, therefore, to lead their arm toward the body. In other words, harder movements can better differentiate the level of impairment of patient and, therefore, can show higher correlations with the clinical scales. With respect to the ICC analysis, the lower value we found for the Length_3_ can be mainly related to the lower variance between patients. Referring to the discriminant ability, it should be underlined that all the robotic indices but the Work_tot_ were significantly different between patients with sub-acute stroke and healthy subjects, with a strong effect size (a moderate effect size was observed only for Work_tan_). With respect to the Duration, our results are in accordance to those obtained, for example, by Otaka et al. [7], or Coderre et al. [13], where higher time necessary to complete planar task were detected in patients with stroke, when compared to healthy subjects. Similarly, with respect to the Velocity_mean_, a reduction of speed in patients with stroke was detected in several studies [6, 12].

A statistically significant difference between the two groups was also found for all the Length and Score parameters, that are related to the ability of the patients to travel the distance toward the target with the impaired arm. Usually these parameters are not assessed in point-to-point reaching tasks performed in a transversal plan, since the patient’s ability to reach the target is a mandatory requirement to be included in the evaluation (see, for example, Otaka et al. [7]). However, a decreased movement distance in reaching task is evident in patients with stroke [44] and, therefore, in our opinion, an evaluation of this aspect could add meaningful information about the patient’s dexterity and the course of the therapy.

Finally, referring to the work-related parameters, to the best of our knowledge, ours is the first study that evaluates the differences between patients with stroke and healthy subject with similar metrics. We found that Work_tan_ was significantly different between the two groups, while the Work_tot_ was not. Zollo et al. [12] employed both total and useful work (similar to the Work_tan_), to assess the effect of the rehabilitation intervention, rather than the motor skills of patients with stroke. Interestingly, Zollo et al. found that the total work did not change after therapy while the useful work increased after the robotic treatment. Their results, along with us, suggest to employ only the useful work, i.e. the work spent to move towards the target, rather than the total work, as a work-related measure of motor impairment in patients with stroke. In our opinion, the Work_tot_ did not differ between stroke patients and healthy individuals because it counts the entire work performed by the subject; with respect to the patients it takes into account the work done to move the robot in a curved path, considering both the “physiological part of the movement” (toward the target) and the “pathological part of the movement” (perpendicular to the correct direction). Therefore, it is combined by two factors, one reducing because of the impairment, and one increasing because of the impairment. This could also affect the correlations with the clinical scales.

A limitation of this study is the absence of robotic measurement assessing movement smoothness. In fact, movement smoothness, quantified by means of several parameters based on velocity or more commonly jerk, was found to be an hallmark of severity in patients with stroke [37]. It is worth noting that, almost the totality of the studies obtained these parameters after a data reduction, starting from the raw data provided by the robot. MOTORE, as well as providing the investigated parameters, allow the access to raw data, and, therefore, allow to compute smoothness parameter. Obviously, this is more time-consuming, and likely, more suitable for use in research rather than in clinical practice. Since this study is especially designed to assess the properties of the provided robotic indices for a routine clinical use, we decided not to consider indices computed from raw data. In fact, the goal of this study is to use these measures to obtain a frequent evaluation during the treatment, with the aim of calibrating the treatment on patient’s needs, ability, and motor changes, in order to design patient-tailored rehabilitation programs. Future work should be addressed to analyze the properties of the measure of smoothness, obtained from raw data.

Finally, the design of this study is cross-sectional. A longitudinal design is needed to measure responsiveness of the robotic parameters after rehabilitation.

## Conclusion

We found that all the robotic indices but the Work_tot_ provided by a novel robotic device for the upper limb rehabilitation, are reliable, sensitive and strongly correlated both with motor and disability clinical scales. Therefore, they are suitable as an evaluation tool for the upper limb motor performance of patients with sub-acute stroke in clinical practice. The instrumental outcome measures are very important to have an objective but also easy evaluation, as well as to define the best treatment for the patient. In fact, the recovery of the upper limb can vary greatly from patient to patient and in this perspective, instrumental and objective data could be a guide to address the treatment path.

## Additional file

Additional file 1: **Table S1.** Correlation between the robotic indices. (DOCX 19 kb)

## Abbreviations

ARAT: Action research arm test
BI: Barthel index
ES: Effect size
FMA-UL: Upper-limb subscale of the Fugl-Meyer Assessment of Motor Recovery after Stroke
ICC: Intraclass correlation coefficient
MOTORE: MObile roboT for upper limb neurOrtho Rehabilitation
