# Supplementary material for: Reliability, validity and discriminant ability of the instrumental indices provided by a novel planar robotic device for upper limb rehabilitation
Source: J Neuroeng Rehabil. 2018 May 16;15:39. doi: 10.1186/s12984-018-0385-8 (PMC5956822; doi:10.1186/s12984-018-0385-8)
Supplement: Supplementary file 1 — Table S1. Correlation between the robotic indices. (DOCX 19 kb) [file 12984_2018_385_MOESM1_ESM.docx]

Table S1 – Correlation between the robotic indices

|  | **Duration** | **Velocity_avg_** | **Length_tot_** | **Score** | **Work_tot_** | **Work_tan_** | **Length_1_** | **Length_2_** | **Length_3_** | **Length_4_** | **Length_5_** | **Length_6_** | **Length_7_** | **Length_8_** |
| --- | --- | --- | --- | --- | --- | --- | --- | --- | --- | --- | --- | --- | --- | --- |
| **Duration** | 1 | -0,800** | -0,835** | -0,915** | 0,051 | -0,750** | -0,730** | -0,537** | -0,523** | -0,612** | -0,788** | -0,806** | -0,877** | -0,827** |
| **Velocity_avg_** | -0,800** | 1 | 0,712** | 0,703** | 0,333* | 0,838** | 0,612** | 0,483** | 0,418** | 0,582** | 0,651** | 0,680** | 0,683** | 0,680** |
| **Length_tot_** | -0,835** | 0,712** | 1 | 0,929** | 0,081 | 0,760** | 0,895** | 0,677** | 0,562** | 0,814** | 0,939** | 0,937** | 0,896** | 0,940** |
| **Score** | -0,915** | 0,703** | 0,929** | 1 | -0,033 | 0,703** | 0,820** | 0,552** | 0,590** | 0,741** | 0,890** | 0,885** | 0,949** | 0,893** |
| **Work_tot_** | 0,051 | 0,333* | 0,081 | -0,033 | 1,000 | 0,431** | 0,004 | 0,109 | 0,131 | 0,228 | -0,019 | 0,026 | 0,001 | 0,072 |
| **Work_tan_** | -0,750** | 0,838** | 0,760** | 0,703** | 0,431** | 1,000 | 0,664** | 0,543** | 0,412** | 0,637** | 0,679** | 0,702** | 0,680** | 0,732** |
| **Length_1_** | -0,730** | 0,612** | 0,895** | 0,820** | 0,004 | 0,664** | 1 | 0,676** | 0,384** | 0,608** | 0,789** | 0,767** | 0,778** | 0,867** |
| **Length_2_** | -0,537** | 0,483** | 0,677** | 0,552** | 0,109 | 0,543** | 0,676** | 1 | 0,308* | 0,305* | 0,501** | 0,544** | 0,513** | 0,623** |
| **Length_3_** | -0,523** | 0,418** | 0,562** | 0,590** | 0,131 | 0,412** | 0,384** | 0,308* | 1 | 0,646** | 0,478** | 0,460** | 0,477** | 0,512** |
| **Length_4_** | -0,612** | 0,582** | 0,814** | 0,741** | 0,228 | 0,637** | 0,608** | 0,305* | 0,646** | 1 | 0,816** | 0,737** | 0,670** | 0,691** |
| **Length_5_** | -0,788** | 0,651** | 0,939** | 0,890** | -0,019 | 0,679** | 0,789** | 0,501** | 0,478** | 0,816** | 1 | 0,936** | 0,848** | 0,823** |
| **Length_6_** | -0,806** | 0,680** | 0,937** | 0,885** | 0,026 | 0,702** | 0,767** | 0,544** | 0,460** | 0,737** | 0,936** | 1 | 0,882** | 0,873** |
| **Length_7_** | -0,877** | 0,683** | 0,896** | 0,949** | 0,001 | 0,680** | 0,778** | 0,513** | 0,477** | 0,670** | 0,848** | 0,882** | 1 | 0,915** |
| **Length_8_** | -0,827** | 0,680** | 0,940** | 0,893** | 0,072 | 0,732** | 0,867** | 0,623** | 0,512** | 0,691** | 0,823** | 0,873** | 0,915** | 1 |

Spearman’s correlation coefficient between the robotic indices . The symbols * and ** indicate a p value less than 0.05 and 0.01 respectively.
